# Characterization of Two Mannitol-Producing *Leuconostoc* Strains from Pa-Kimchi and Their Application for Juice and Yogurt Fermentation

**DOI:** 10.4014/jmb.2301.01015

**Published:** 2023-02-13

**Authors:** Yun Ji Kang, Min Jae Kim, Tae Jin Kim, Jeong Hwan Kim

**Affiliations:** 1 Division of Applied Life Science (BK21 Four), Graduate School, Gyeongsang National University, Jinju 52828, Republic of Korea; 2 Institute of Agriculture and Life Science, Gyeongsang National University, Jinju 52828, Republic of Korea

**Keywords:** Mannitol, mannitol dehydrogenase, *Leuconostoc*, shine muscat juice, yogurt

## Abstract

Two mannitol producing lactic acid bacteria were isolated from pa (green onion)- kimchi, identified and named as *Leuconostoc mesenteroides* SKP 88 and *Leuconostoc citreum* SKP 92, respectively. Both isolates grew well at 25-30°C, initial pH 6-8, and 3% and lower NaCl concentration. Both isolates converted fructose into mannitol efficiently when grown on MRS broth containing fructose and glucose. Glucose was used as a carbon source and fructose was used as a precursor for mannitol. Mannitol yields were the highest in MRS broth with 3% fructose and 2% glucose. Shine muscat juice fermentation was done using each isolate as a starter. As fermentation progressed, decrease in pH and increases in titratable acidity and viable counts were observed. *L. mesenteroides* SKP 88 showed better mannitol conversion ability than *L. citreum* SKP 92, and shine muscat juice fermented with *L. mesenteroides* SKP 88 showed the mannitol production of 41.6 g/l at 48 h, and juice fermented with *L. citreum* SKP 92 showed 23.4 g/l at the same time. Yogurt fermentations showed similar patterns, and yogurt fermented with *L. mesenteroides* SKP 88 showed the mannitol production of 15.13 g/l. These results showed that both strains are useful as starters for healthy fermented foods with reduced fructose contents.

## Introduction

Mannitol is a 6 carbon sugar alcohol naturally present among microorganisms and plants [1]. Mannitol confers cooling sweetness to foods and receives increasing interests as an alternative sweetner replacing traditional sweetners such as sucrose and fructose due to its low glycemic index and calorie value [2, 3]. Mannitol is less cariogenic and absorbed slowly in intestinal tracts of human and thus does not increase blood sugar level significantly. Therefore, mannitol is an ideal sweetner for diabetics and people in diet [4, 5]. Mannitol can be produced from sucrose via 3 different ways. Chemical synthesis is the most commonly used where fructose in glucose/fructose mixture is reduced into mannitol by a metal catalyst and hydrogen gas at high temperature and high pressure [2, 6]. But chemical method requires complex purification steps to remove side products such as sorbitol and metal catalyst. Second method is enzymatic reduction method where fructose is reduced into mannitol by mannitol dehydrogenase. But this method requires an expensive cofactor, NADH, and highly purified enzyme [6]. Considering these shortcomings, microbial fermentation seems the most promising method for mannitol production. Mannitol is produced through fermentation among many fungi, yeasts and bacteria [7, 8]. Among microorganisms, lactic acid bacteria (LAB) are the most preferred hosts for mannitol production because many LAB transform fructose into mannitol efficiently in addition to the GRAS status and other health-promoting effects [9]. Two different pathways exist for mannitol production among LAB. In heterolactic fermenters such as *Leuconostocs* species, fructose is reduced into mannitol by mannitol dehydrogenase. In homolactic fermenters such as Lactococcus species, mannitol is produced from fructose 6-phosphate, which is first reduced into mannitol 1-phosphate by mannitol 1-phosphate dehydrogenase followed by dephosphorylation by mannitol 1-phosphatase [9].

Metabolic engineering works have been reported for *Leuconostoc* species to increase mannitol production yield, which included inactivation of a fructokinase gene converting fructose into fructose 6-phosphate together with inactivation of a lactate dehydrogenase gene [10]. For *Lactococcus lactis*, a homolactic fermenter, mutants in PEP-PTS system for mannitol transport were constructed for increasing mannitol production [11]. LAB strains with increased mannitol production are very useful as starters for fermented foods which contain reduced fructose contents. Such fermented foods contain less calorie when comsumed and could be considered healthier and more functional than foods containing more fructose [12].

In this study, two LAB strains efficiently converting fructose into mannitol were isolated from pa (green onion)-kimchi, and identified as *Leuconostoc mesenteroides* and *Leuconostoc citreum*, respectively. Their growth properties in media containing fructose and glucose were studied and both isolates were used as starters for shine muscat juice and yogurt fermentations. Mannitol production from fructose was confirmed, indicating the potentials of both strains as starters for fermented foods with reduced fructose contents.

## Materials and Methods

### Isolation of Mannitol-Producing LAB from Pa-Kimchi

Pa-kimchi was purchased at a local market at Sacheon, Gyeongnam, republic of Korea in July, 2020, and homogenized by using stomacher 80 (Seward, UK) with 0.1% peptone water. Serially diluted homogenates were spreaded onto de Man, Rogosa, and Sharpe (MRS, Becton Dickinson Co., USA) agar plates with 1% CaCO_3_ and 0.006% bromocresol purple [13]. Plates were incubated for 48 h at 30°C. Colonies with yellow color and surrounded with clear zone were selected as putative LAB and tested for mannitol production. Isolates were grown in MRS broth with 2% fructose (no glucose) for 24 h at 30°C. Cultures were centrifuged (12,000 ×*g* for 3 min), and supernatants were analyzed by thin-layer chromatography (TLC) using TLC plates (Merck, # 1057150001, Germany). A solvent mixture of acetonitrile: ethyl acetate: 1-propanol: water (85:20:20:15, v/v/v/v) was used for separation. The plate was dipped in AgNO3-acetone for 5 min and in alkaline-methanol for 2 min [14].

### Identification of Mannitol-Producing Isolates

Mannitol-producing isolates were identified by 16S rRNA genes sequencing. A DNA purification kit (Cosmogenetech, Korea) was used to extract genomic DNA. 16S rRNA genes were amplified using primer 27F (5’-AGAGTTTGATCMTGGCTCAG-3’) and 1492R (5’-TACGGYTACCTTGTTACGACTT-3’) [15]. PCR was done using a MJ mini thermal cycler (BioRad, USA), and the reaction mixture (50 ml) consisted of 5 μl DNA (100 ng), 0.5 μl (1 U) Ex *Taq* DNA polymerase (Takara, Japan), 5 μl 10 X buffer, 5 μl dNTP mixture (2.5 mM each), 1 μl each primer (10 pmol), and 32.5 μl distilled water. Amplification conditions were as follows; 94°C for 5 min followed by 30 cycles of 94°C for 30 S, 58°C for 45 S, 72°C for 1 min, and a final extension at 72°C for 4 min [13]. PCR products were purified using a purification kit (Favorgen, Taiwan). DNA sequencing was done and the nucleotide sequences were analyzed by Basic Local Alignment Search Tool (BLAST) at National Center for Biotechnology Information (NCBI, USA).

### Growth of Mannitol-Producing Isolates

Growth properties of two isolates were examined in MRS broth under different conditions (temperature, initial pH and NaCl content). Each strain was first propagated in 10 ml MRS broth for 24 h at 30°C, then inoculated into fresh MRS broth (1%, v/v), and cultivated at 4-45°C without shaking for 72 h. Each strain was inoculated into MRS broth where the initial pH was adjusted to 4, 5, 6, 7, or 8, respectively. Each strain was inoculated into MRS broth with NaCl (0-7%, w/v). Growth was monitored by measuring absorbance at 600 nm (UV-1601, Shimadzu, Japan).

### Stress Resistance of Mannitol-Producing Isolates

Two isolates were examined for their resistances against low pH and bile salts by the methods described previously [16]. Two isolates were cultivated in MRS broth until the OD_600_ reached 1.5. One ml of culture was centrifuged at 12,000 ×*g* for 5 min at 4 ° and cell pellet was resuspended in MRS broth where the pH was adjusted to 2, 3, 4 or 6.5 (control), respectively. Cell suspensions were incubated for 2 h at 30°C, and then viable cells were counted by standard plate method. Cells grown overnight in MRS broth were harvested by centrifugation, washed and resuspended in MRS broth (1 ml) supplemented with 0.3% bile salts (Fluka, USA). Viable cells were counted after 2 h incubation at 30°C.

### Mannitol Yields according to Sugar Contents

Two isolates were cultivated for 24 h at 30°C in MRS broth with different fructose and glucose contents: MRS with 5% fructose, MRS with 3% fructose, MRS with 5% fructose and 2% glucose, and MRS with 3% fructose and 2% glucose. Supernatants were obtained by centrifugation (9,950 ×*g*, 10 min) and analyzed by gas chromatography (GC-2010 Plus, GCMS-TQ 8030, Shimazu, Japan) employing an DB-5 MS column (30 m × 0.25 mm id, 0.25 mm film thickness, J & W scientific, USA) as described previously [17]. Mannitol yield was calculated from the conversion ratio of fructose to mannitol.

Mannitol production yield = amount of mannitol produced/amount of fructose consumed (w/w).

### Shine Muscat Juice Fermentation

Shine muscat juice was prepared by crushing shine muscat grapes harvested in July, 2022 (Kyungsan, Korea) using a blender and the homogenate was centrifuged (5,000 ×*g*, 20 min, 4°C) to remove particulates. The juice was supplemented with tween 80 (1 ml/l) and yeast extract (2 g/l), and pH was adjusted to 6.5 with 1 N NaOH, and sterilized at 110°C for 10 min [18]. Shine muscat juice was inoculated with *L. mesenteroides* SKP 88 or *L. citreum* SKP 92 (10^7^ CFU/ml) and fermented for 48 h at 30°C.

Juice samples were collected at 0, 12, 24, 36, and 48 h of fermentation, and pH, titratable acidity (TA), viable cell counts, and sugar (mannitol, glucose, fructose) contents were analyzed. pH was measured using a pH meter (Thermo Fisher, USA), and TA was calculated by titration of juice supernatant with 0.1 N NaOH to pH 8.4. The amount of added NaOH was used to calculate the amount of lactic acid (%). Viable cells were counted by standard plate method using MRS agar plates. Juice samples were centrifuged at each time point, and the supernatants were analyzed by TLC as described above. Sugar contents were analyzed using high-performance liquid chromatography (HPLC, 1260 series, Agilent Com., USA) employing an Agilent Zorbax carbohydrate column (4.6 mm × 150 mm, i.d, 5 μm), and an RI (refractive index) detector. Water was used as the mobile phase at 1.4 ml/min.

### Yogurt Fermentation

Reconstituted milk was prepared by dissolving whole milk powder (Seoul dairy cooperative, Korea) in distilled water (11.5%, w/v). Reconstituted milk was fortified with fructose (3%) and glucose (2%) and heat treated at 90°C for 10 min. Two most common yogurt starters, *Streptococcus thermophilus* and *Lactobacillus delbrueckii* ssp. *bulgaricus*, were obtained from National Institute of Agricultural Sciences (Wanju, Jeonbuk, Korea) and used as starters for control yogurt. Five yogurt samples were prepared: yogurt prepared with *S. thermophilus* and *L. delbrueckii* ssp. *bulgaricus* (control, yogurt 1), yogurts prepared with two common cultures plus a single culture of *L. mesenteroides* SKP 88 (yogurt 2) or *L. citreum* SKP 92 (yogurt 3), and yogurts prepared with a single culture of *L. mesenteroides* SKP 88 (yogurt 4) or *L. citreum* SKP 92 (yogurt 5), Yogurts were inoculated with starters (5 × 10^6^ CFU/ml), and fermented for 24 h at 37°C. After fermentation, yogurts were stabilized for 24 h at 10°C, and then stored for 24 h at 4°C.

Yogurt samples were collected at 0, 6, 12, 24 h of fermentation, 48 h after stabilization, and 72 h after storage. pH, TA, viable cell counts, and sugar contents (mannitol, glucose, and fructose contents) were measured as described above. Mannitol, glucose, and fructose contents were measured from 72 h samples by HPLC as described above. Syneresis was determined as follows: 10 g of yogurt sample was spread across a Whatman No. 1 filter paper as a thin layer to cover the surface, and filtered for 10 min. The quantity of liquid that passed through the filter paper was measured. Syneresis (%) was expressed as weight of drained whey per weight of yogurt (10 g) × 100 [19, 20].

### Statistical Analysis

All data were expressed as the mean ± standard deviation of triplicate experiments. All data obtained from measurements were evaluated by ANOVA in SAS, ver. 3.8 (SAS Institute Inc., USA). Significance of a difference between the means of measured values was analyzed by Duncan’s multiple range test (*p* < 0.05).

## Results and Discussion

### Isolation of Mannitol-producing LAB from Kimchi

**Fig. 1:**
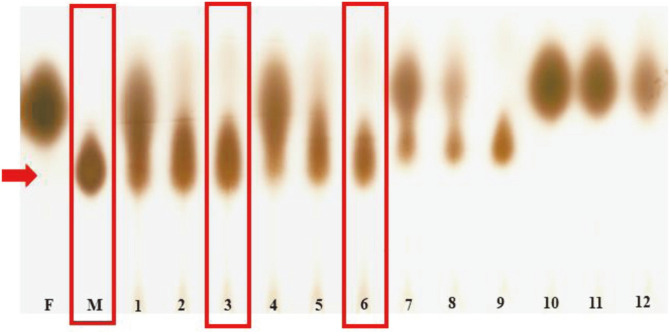
Thin-layer chromatogram showing mannitol production from fructose. F, 2% fructose; M, 1% mannitol; lanes 1-3, SKP 88 at 6 h (1), 12 h (2), 24 h (3); lanes 4-6, SKP 92 at 6 h (4), 12 h (5), 24 h (6); lanes 7-9, SKP 100 (an isolate producing mannitol weakly) at 6 h (7), 12 h (8), 24 h (9); lanes 10-12, SKP 122 (an isolate non-producing mannitol) at 6 h (10), 12 h (11), 24 h (12). One ml of standard or culture supernatant was spotted on a TLC plate.

A total of 500 LAB isolates were obtained from fermented foods and two isolates, SKP 88 and SKP 92, from pa (green onion)-kimchi showed efficient mannitol production from fructose when analyzed by TLC (Fig. 1). 16S rRNA gene sequencing identified SKP 88 as *Leuconostoc mesenteroides*, and SKP 92 as *Leuconostoc citreum*. The Genbank accession numbers of 16S rRNA genes are ON651647 and ON651649 for SKP 88 and SKP 92, respectively.

### Growth Properties of *L. mesenteroides* SKP 88 and *L. citreum* SKP 92

**Fig. 2:**
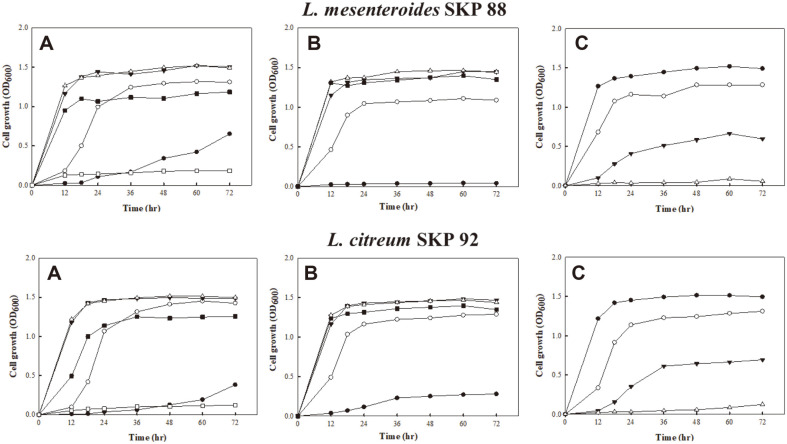
Growth of two isolates on MRS broth under different conditions. (**A**) temperature: ●, 4°C; ○, 15°C; ▼, 25°C; △, 30°C; ■, 37°C; □, 45°C. (**B**) initial pH: ●, pH 4; ○, pH 5; ▼, pH 6; △, pH 7; ■, pH 8. (**C**) NaCl content: ●, 0%; ○, 3%; ▼, 5%; △, 7%.

*L. mesenteroides* SKP 88 and *L. citreum* SKP 92 were cultivated for 72 h under different conditions (Fig. 2). Both strains grew rapidly at 25 and 30°C, slowly at 4, 15, and 37°C, and did not grow at 45°C. The optimum growth temperatures were 25 and 30°C, and the highest cell numbers (OD_600_ = 1.4 - 1.5) reached in 24 h. Both strains showed the optimum growth at the initial pH of 6.0 and 7.0, and growth was retarded at lower pH. *L. citreum* SKP 92 grew slowly but *L. mesenteroides* SKP 88 did not grow at pH 4.0. Both strains grew well at 3% and lower NaCl concentrations.

### Stress Resistance of Mannitol-Producing Isolates

The ability of an organism to survive under low pH environments is an important requirement for being a probiotic. *L. mesenteroides* SKP 88 showed higher than 50% and lower than 20% viabilities after 2 h exposure to pH 4.0 and pH 3.0, respectively at 30°C (Table 1). *L. citreum* SKP 92 showed higher than 50% viabilities at pH 3.0 and pH 4.0. When exposed to pH 2.0 for 2 h at 30°C, the survival ratios of both strains decreased to 0%. However, *L. citreum* SKP 92 survived better than *L. mesenteroides* SKP 88. After exposure to 0.3% bile salt for 2 h at 30°C, survival ratios were 31.48% and 61.61% for *L. mesenteroides* SKP 88 and *L. citreum* SKP 92, respectively (Table 2). Survival ratios were further decreased to 9.27% and 55.77% for *L. mesenteroides* SKP 88 and *L. citreum* SKP 92, respectively, when both strains were first exposed to pH 3 for 2 h and then exposed to 0.3% bile salt for 2 h (Table 2). Considering these results, *L. citreum* SKP 92 could be used as a probiotic.

### Mannitol Yields according to Fructose/Glucose Contents

The amounts and the ratios between fructose and glucose in MRS broth affected the mannitol production yields of two strains (Table 3). Both strains produced more mannitol in MRS broth with 5% fructose than in MRS broth with 3% fructose. Both strains produced more mannitol in MRS broth with 3% fructose and 2% glucose than in MRS broth with 3% fructose only. *L. mesenteroides* SKP 88 produced the highest amount of mannitol in MRS broth with 5% fructose (no glucose) but *L. citreum* SKP 92 produced the highest amount of mannitol in MRS broth with 5% fructose and 2% glucose. Mannitol yield was increased by the co-presence of glucose and fructose, and the highest yields were observed in MRS broth with 3% fructose and 2% glucose for both strains. In MRS broth with 3% fructose and 2% glucose, *L. mesenteroides* SKP 88 consumed 27 g fructose and produced 28.7 g mannitol with the mannitol yield of 105.14 mol%. *L. citreum* SKP 92 consumed 25.2 g fructose and produced 24.8 g mannitol with the mannitol yield of 97.32 mol%. Both strains metabolized glucose first as a carbon and energy source and reduced most fructose into mannitol [9]. The mannitol yields of both strains were decreased in MRS broth with 5% fructose and 2% glucose although more fructose was consumed together with more mannitol produced. *L. citreum* SKP 92 showed more decrease in mannitol yield than *L. mesenteroides* SKP 88, reflecting differences in sugar metabolisms of two species. Some fructose might be metabolized as a carbon source rather than precursor of mannitol after glucose was depleted. *L. mesenteroides* SKP 88 showed more than 100% mannitol yield in MRS broth with fructose and glucose. It was suspected that *L. mesenteroides* SKP 88 might be able to convert some glucose into mannitol via pathways interconnecting fructose and glucose catabolism [9]. The presence of other pathways where NADH is oxidized into NAD^+^ or vice versa was likely to affect the reduction of fructose into mannitol [21, 22]. Only few combinations of glucose and fructose were tested in this study, and it is necessary to check growth and mannitol conversion of two strains under more diverse combinations of fructose and glucose. Overall, *L. mesenteroides* SKP 88 showed excellent mannitol yield, more than 100% yield in MRS broth containing 3% or 5% fructose and 2% glucose.

Mannitol production yield of *Leuconostoc mesenteroides* NRRL B-1149 was 78 mol% when grown in LM medium (yeast extract, 5 g/l; peptone, 5 g/l; K_2_HPO_4_, 2 g/l; MgSO_4_ 7H_2_O, 0.2 g/l; NaCl, 0.01 g/l; FeSO_4_, 0.01 g/l; MnSO_4_ H_2_O, 0.01 g/l; CaCl_2_ 2H_2_O, 0.015 g/l) with 5% fructose without aeration at pH 5.0 and 28°C [14]. Mannitol yield of *Leuconostoc citreum* KACC 91348P was 86.6 mol% when grown in simplified medium (tryptone, 10 g/l; yeast extract, 5 g/l; K_2_HPO_4_, 2 g/l; MgSO_4_, 0.2 g/l; MnSO_4_, 0.01 g/l; CaCl_2_, 0.02 g/l; NaCl, 0.02 g/l) with 3% fructose and 1.5% glucose at pH 6.5 and 30°C [23], whereas the mannitol yield of *Leuconostoc pseudomesenteroides* ATCC12291 was 73.7 mol% when grown in SP medium (tryptone, 10 g/l; yeast extract, 5 g/l; K_2_HPO_4_, 2 g/l; MgSO_4_, 0.2 g/l; MnSO_4_, 0.01 g/l; CaCl_2_, 0.02 g/l; NaCl, 0.01 g/l; FeCl_3_, 0.01 g/l) with 2% fructose and 1% glucose at pH 6.2 and 30°C [10]. It was not possible, however, to compare the mannitol production yields of previous reports with those of *L. mesenteroides* SKP 88 and *L. citreum* SKP 92 because previous works were done using bioreactors and growth conditions including culture media, pH, temperature, aeration, and the stage of growth were all different from each other. Based on our results, it could be concluded that *L. mesenteroides* SKP 88 and *L. citreum* SKP 92 convert fructose into mannitol efficiently like other reported LAB, and both strains are useful as starters for production of healthy drinks and other fermented foods with reduced fructose contents.

### Shine Muscat Juice Fermentation

**Fig. 3:**
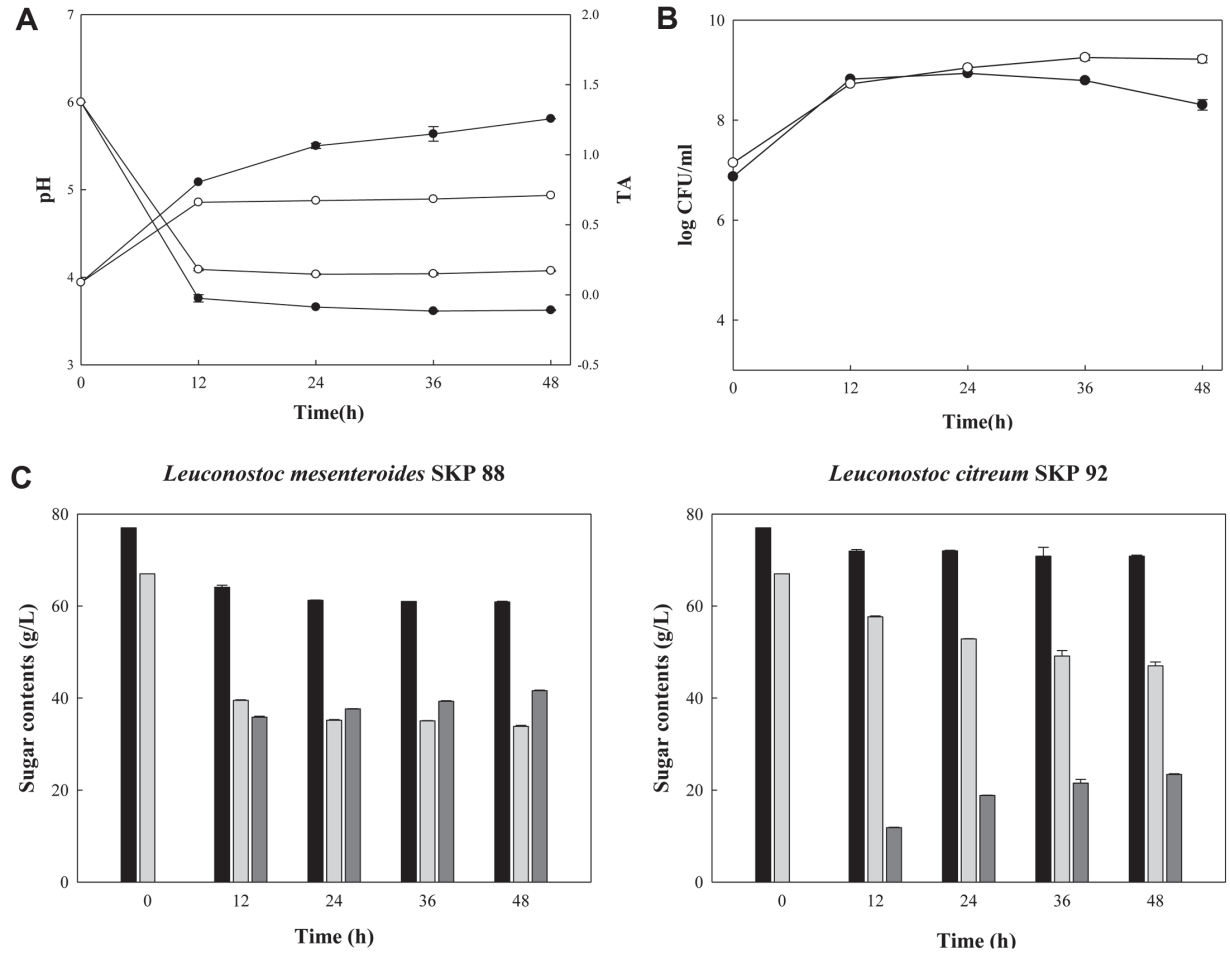
pH, TA, LAB counts and sugar contents of shine muscat juice samples during fermentation. (**A**) pH and TA, (**B**) LAB counts, (**C**) sugar contents. ●, shine muscat juice fermented with *L. mesenteroides* SKP 88; ○, shine muscat juice fermented with *L. citreum* SKP 92. Black, gray, and dark gray bars represent glucose, fructose, and mannitol contents, respectively.

pH of all juice samples decreased during fermentation (Fig. 3A). *L. mesenteroides* SKP 88 and *L. citreum* SKP 92, heterolactic fermenters, produced lactic acid and acetic acid from glucose and fructose during fermentation, lowering pH of juice samples. The pH of shine muscat juice fermented with *L. mesenteroides* SKP 88 was 3.62 ± 0.01 at 48 h, lower than that of juice fermented with *L. citreum* SKP 92. TA increased with fermentation time, and a significant increase occurred between 0 and 12 h, similar to pH changes but in a reverse direction (Fig. 3A). TA of juice fermented with *L. mesenteroides* SKP 88 reached the highest value of 1.38 ± 0.02 at 48 h, and TA of juice fermented with *L. citreum* SKP 92 was 0.73 ± 0.04 at 48 h.

LAB counts increased from 10^7^ CFU/ml (time 0) to 8.65 × 10^8^ CFU/ml and 1.67 × 10^9^ CFU/ml for juice fermented with *L. mesenteroides* SKP 88 and *L. citreum* SKP 92, respectively, at 24 h of fermentation (Fig. 3B). LAB counts of juice fermented with *L. mesenteroides* SKP 88 were lower than those of juice fermented with *L. citreum* SKP 92. It was suspected that *L. mesenteroides* SKP 88 produced more acid, which caused lower pH of juice and rapid death of *L. mesenteroides* SKP 88 cells.

*L. mesenteroides* SKP 88 produced more mannitol than *L. citreum* SKP 92 during juice fermentation as judged by TLC (results not shown), consistant with the results from growth in MRS broth with fructose and glucose. Sugar contents of juice samples were analyzed by HPLC (Fig. 3C). Glucose content of juice fermented with *L. mesenteroides* SKP 88 decreased rapidly during the first 12 h and then decreased slowly until 24 h. After 24 h, glucose content remained stably. Fructose contents of juice samples decreased in a similar pattern. Glucose and fructose contents of juice fermented with *L. citreum* SKP 92 decreased less. *L. mesenteroides* SKP 88 consumed 33.15 g/l (49.5%) fructose and 16.1 g/l (20.9%) glucose at 48 h of fermentation, and produced 41.6 g/l mannitol at the same time. *L. citreum* SKP 92 consumed 20 g/l (29.9%) fructose and 6.15 g/l (8.0%) glucose, and produced 23.4 g/l mannitol after 48 h of fermentation.

### Yogurt Fermentation

**Fig. 4:**
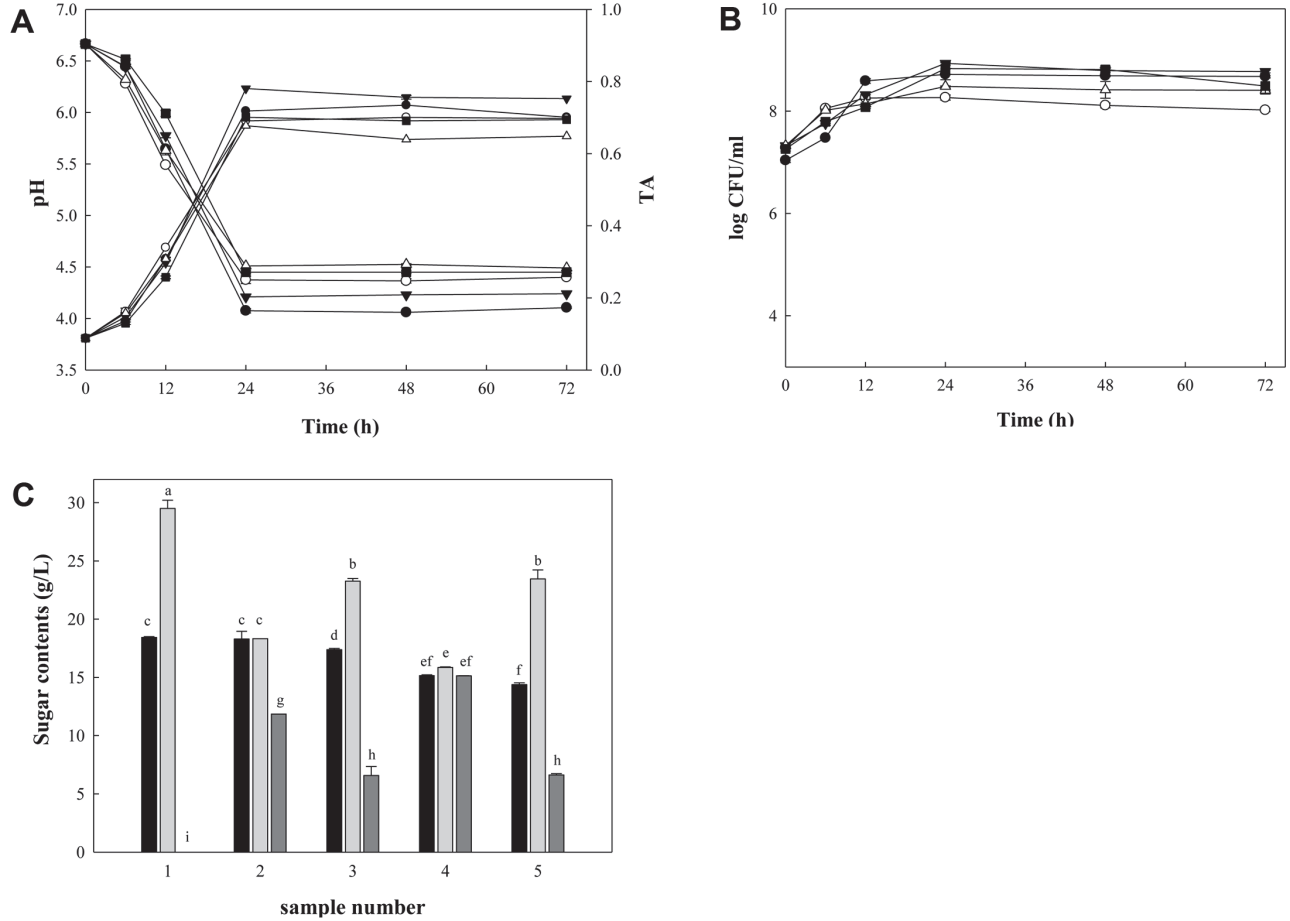
pH, TA, LAB counts and sugar contents of yogurts during fermentation. (**A**) pH and TA, (**B**) LAB counts, (**C**) sugar contents. ●, yogurt 1 (*S. thermophilus* and *L. bulgaricus*); ○, yogurt 2 (*S. thermophilus*, *L. bulgaricus*, and *L. mesenteroides* SKP 88); ▼, yogurt 3 (*S. thermophilus*, *L. bulgaricus*, and *L. citreum* SKP 92); △, yogurt 4 (*L. mesenteroides* SKP 88); ■, yogurt 5 (*L. citreum* SKP 92). Black, gray, and dark gray bars represent glucose, fructose, and mannitol contents, respectively.

pH of all yogurt samples decreased during fermentation (Fig. 4A). The pH of yogurt 1 (*S. thermophilus* and *Lb. delbrueckii* ssp. *bulgaricus*) was 4.07 ± 0.02 at 24 h. The pH of yogurt 2 was 4.37 ± 0.04 and that of yogurt 3 was 4.21± 0.00 at 24 h. Yogurt 4 and 5 showed higher pH values (4.51 ± 0.00, 4.45 ± 0.01). During the storage period, pH of yogurt samples remained constant. TA increased with fermentation time, and a significant increase occurred between 0 and 24 h, similar to pH changes but in a reverse direction (Fig. 4A). Yogurt 3 (2 commercial starters plus *L. citreum* SKP 92) showed the highest TA (0.78 ± 0.00) at 48 h, whereas yogurt 4 (*L. mesenteroides* SKP 88) showed the lowest TA (0.68 ± 0.00). TA changes of yogurt samples were different from those of shine muscat juice samples where juice fermented with *L. mesenteroides* SKP 88 showed higher TA than juice fermented with *L. citreum* SKP 92. The difference might be caused by co-cultivation with 2 common yogurt starters. After fermentation, TA remained nearly constant.

The initial LAB count was 10^7^ CFU/ml, and the number increased during 24 h of fermentation at 37°C (Fig. 4B). Yogurt 5 (*L. citreum* SKP 92) showed the highest LAB count (6.78 × 10^8^ CFU/ml) at 24 h, whereas yogurt 2 (2 commercial yogurt starters plus *L. mesenteroides* SKP 88) showed the lowest count (1.84 × 10^8^ CFU/ml). After fermentation, LAB counts of all yogurt samples remained nearly constant.

After 1 day storage at 4°C (72 h), syneresis of yogurt 1 (control, 39.2 ± 0.64%) was lower than those of yogurt 4 (48.5 ± 1.41%) and 5 (51 ± 1.41%). Syneresis of yogurt 2 (39 ± 0.85%) and yogurt 3 (41.13 ± 0.18%) were also lower than those of yogurt 4 and 5. Further studies are necessary to improve the texture of yogurt fermented with either *L. mesenteroides* SKP 88 or *L. citreum* SKP 92.

Yogurt 2 and 4 showed higher mannitol production yields than yogurt 3 and 5 by TLC (results not shown), indicating higher mannitol production ability of *L. mesenteroides* SKP 88. TLC and HPLC results (Fig. 4C) confirmed mannitol production from fructose by two strains during yogurt fermentation. Skim milk powder used contained significant amount of lactose (and other sugars). There was no description on the composition of sugars of skim milk powder except total amount of sugars. 49 g sugars/100 g powder. Lactose was most likely used as a carbon source, especially by two common yogurt starters [24]. This explained the small reduction of glucose contents in yogurt samples. *L. mesenteroides* SKP 88 and *L. citreum* SKP 92 utilized more glucose than two yogurt starters for growth. Unlike commercial yogurt starters, *L. mesenteroides* SKP 88 and *L. citreum* SKP 92 seemed to prefer glucose for growth. Yogurt 4 (*L. mesenteroides* SKP 88) showed the highest mannitol production of 15.13 g/l, whereas yogurt 3 (2 commercial yogurt starters plus *L. citreum* SKP 92) showed mannitol production of 6.58 g/l. Mannitol wasn’t observed in yogurt 1 (2 commercial starters), indicating mannitol production was an unique property of a strain.

Heterolactic fermenters such as *Leuconostoc* species convert fructose into mannitol by mannitol dehydrogenase and NADH [9]. If significant amount of fructose in foods is converted to mannitol, the foods become healthier because of reduced glycemix index and lower calorie value. Thus *L. mesenteroides* SKP 88 and *L. citreum* SKP 92 have an additional advantage of improving food functionalities in addition to already known advantages [25]. To increase mannitol yield, a gene encoding fructokinase responsible for phosphorylation of fructose into fructose 6-phospahate was inactivated together with inactivation of a *ldh* gene encoding lactate dehydrogenase, encouraging NAD+ generation from NADH via fructose reduction rather than pyruvate reduction [22, 26]. Such metabolic engineering efforts could be applied for *L. mesenteroides* SKP 88 and *L. citreum* SKP 92 to increase mannitol production yields. More detailed studies are also necessary for both strains under various sugar compositions to find out the effect of each sugar content on the growth of each strain.

## Figures and Tables

**Table 1:** Acid tolerance of two mannitol-producing strains.

Strains	pH 6.5^a^ (CFU/ml)	pH 4.0 (CFU/ml)	SR^b^(%)	pH 3.0 (CFU/ml)	SR(%)	pH 2.0 (CFU/ml)	SR(%)
*L. mesenteroides* SKP 88	1.48 × 10^9^	7.87 × 10^8^	53.18	2.73 × 10^8^	18.45	1 × 10^2^	0.000
*L. citreum* SKP 92	3.76 × 10^9^	2.23 × 10^9^	59.31	2.1 × 10^9^	55.85	3.23 × 10^5^	0.009

^a^Control, cells suspended in MRS broth (pH 6.5).

^b^SR (survival ratio), cell numbers in MRS broth (pH 4.0, 3.0 or 2.0)/cell numbers in MRS broth (pH 6.5) × 100.

**Table 2:** Bile acid tolerance of two mannitol-producing strains.

Strains	Control^a^ (CFU/ml)	0.3% bile salts (CFU/ml)	SR^b^(%)	pH 3.0 + 0.3% bile salts^c^ (CFU/ml)	SR^b^(%)
*L. mesenteroides* SKP 88	4.13 × 10^8^	1.3 × 10^8^	31.48	3.83 × 10^7^	9.27
*L. citreum* SKP 92	6.33 × 10^8^	3.9 × 10^8^	61.61	3.53 × 10^8^	55.77

^a^Control, cells in MRS broth (pH 6.5).

^b^SR (survival ratio), cell numbers in MRS broth (0.3% bile salts or pH 3.0 + 0.3% bile salts)/ cell numbers in MRS broth (pH 6.5) × 100

^c^Cells exposed to pH 3 first and then exposed to 0.3% bile salts.

**Table 3:** Mannitol yields according to fructose and glucose contents of culture medium.

Strains	*L. mesenteroides* SKP 88				*L. citreum* SKP 92			
Medium	**3F0G** ^a^	**5F0G** ^b^	**3F2G** ^c^	**5F2G** ^d^	**3F0G**	**5F0G**	**3F2G**	**5F2G**
Fructose consumption (g/l)	30 (± 0.0)	47.99 (± 0.2)	26.96 (± 0.3)	30.76 (± 1.0)	28.56 (± 0.2)	46.39 (± 0.6)	25.21 (± 0.1)	34.23 (± 0.3)
Mannitol production (g/l)	20.78 (± 0.1)	33.58 (± 0.3)	28.70 (± 0.1)	31.94 (± 0.7)	19.39 (± 0.0)	30.43 (± 0.2)	24.83 (± 0.1)	31.2 (± 0.0)
Mannitol yields (Mannitol production/ Fructose consumption) mol%	68.50	69.20	105.14	102.69	67.14	64.87	97.32	90.14

^a^3F0G, MRS broth with 3% fructose (w/v).

^b^5F0G, MRS broth with 5% fructose.

^c^3F2G, MRS broth with 3% fructose and 2% glucose.

^d^5F2G, MRS broth with 5% fructose and 2% glucose.
